# Sustained HIF activation in adult cardiomyocytes show transient beneficial effect in murine HFpEF model

**DOI:** 10.1093/ehjopen/oeaf178

**Published:** 2026-01-22

**Authors:** Daigo Sawaki, Takayuki Isagawa, Shigeru Sato, Tatsuyuki Sato, Hiroaki Semba, Hiroki Sugimoto, Kazutoshi Ono, Ariunbold Chuluun-Erdene, Thuc Toan Pham, Ryohei Tanaka, Toshinaru Kawakami, Masamichi Ito, Shun Minatsuki, Yasutomi Higashikuni, Masataka Asagiri, Ichiro Manabe, Takahide Kohro, Takahiro Kuchimaru, Yasushi Imai, Norihiko Takeda

**Affiliations:** Division of Clinical Pharmacology, Department of Pharmacology, Jichi Medical University, 3311-1 Yakushiji, Shimotsuke, Tochigi, 329-0498, Japan; Division of Cardiology and Metabolism, Centre for Molecular Medicine, 3311-1 Yakushiji, Shimotsuke, Jichi Medical University, Tochigi, 329-0498, Japan; Division of Cardiology and Metabolism, Centre for Molecular Medicine, 3311-1 Yakushiji, Shimotsuke, Jichi Medical University, Tochigi, 329-0498, Japan; Data Science Centre, Jichi Medical University, 3311-1 Yakushiji, Shimotsuke, Tochigi, 329-0498, Japan; Division of Cardiology and Metabolism, Centre for Molecular Medicine, 3311-1 Yakushiji, Shimotsuke, Jichi Medical University, Tochigi, 329-0498, Japan; Division of Cardiology and Metabolism, Centre for Molecular Medicine, 3311-1 Yakushiji, Shimotsuke, Jichi Medical University, Tochigi, 329-0498, Japan; Research Fellow of Japan Society for the Promotion of Science, Tokyo, Japan; Division of Cardiology and Metabolism, Centre for Molecular Medicine, 3311-1 Yakushiji, Shimotsuke, Jichi Medical University, Tochigi, 329-0498, Japan; Division of Cardiology and Metabolism, Centre for Molecular Medicine, 3311-1 Yakushiji, Shimotsuke, Jichi Medical University, Tochigi, 329-0498, Japan; Division of Cardiology and Metabolism, Centre for Molecular Medicine, 3311-1 Yakushiji, Shimotsuke, Jichi Medical University, Tochigi, 329-0498, Japan; Division of Nephrology, Department of Internal Medicine, Jichi Medical University, 3311-1Yakushiji, Shimotsuke, Tochigi, 329-0498, Japan; Division of Cardiology and Metabolism, Centre for Molecular Medicine, 3311-1 Yakushiji, Shimotsuke, Jichi Medical University, Tochigi, 329-0498, Japan; Division of Cardiology and Metabolism, Centre for Molecular Medicine, 3311-1 Yakushiji, Shimotsuke, Jichi Medical University, Tochigi, 329-0498, Japan; Department of Cardiovascular Medicine, Graduate School of Medicine, The University of Tokyo, 7-3-1 Hongo, Bunkyo-ku, Tokyo, 113-8655, Japan; Department of Cardiovascular Medicine, Graduate School of Medicine, The University of Tokyo, 7-3-1 Hongo, Bunkyo-ku, Tokyo, 113-8655, Japan; Department of Cardiovascular Medicine, Graduate School of Medicine, The University of Tokyo, 7-3-1 Hongo, Bunkyo-ku, Tokyo, 113-8655, Japan; Department of Cardiovascular Medicine, Graduate School of Medicine, The University of Tokyo, 7-3-1 Hongo, Bunkyo-ku, Tokyo, 113-8655, Japan; Division of Cardiovascular and Genetic Research, Centre for Molecular Medicine, Jichi Medical University, Tochigi, 329-0498, Japan; Department of Pharmacology, Graduate School of Medicine and RICeD, Yamaguchi University, 1-1-1 Minai-Kogushi, Ube, Yamaguchi, 755-8505, Japan; Department of Systems Medicine, Chiba University Graduate School of Medicine, 1-8-1 Inohana, Chuo-ku, Chiba, 260-8670, Japan; Data Science Centre, Jichi Medical University, 3311-1 Yakushiji, Shimotsuke, Tochigi, 329-0498, Japan; Division of Cardiology and Metabolism, Centre for Molecular Medicine, 3311-1 Yakushiji, Shimotsuke, Jichi Medical University, Tochigi, 329-0498, Japan; Division of Clinical Pharmacology, Department of Pharmacology, Jichi Medical University, 3311-1 Yakushiji, Shimotsuke, Tochigi, 329-0498, Japan; Division of Cardiology and Metabolism, Centre for Molecular Medicine, 3311-1 Yakushiji, Shimotsuke, Jichi Medical University, Tochigi, 329-0498, Japan; Department of Cardiovascular Medicine, Graduate School of Medicine, The University of Tokyo, 7-3-1 Hongo, Bunkyo-ku, Tokyo, 113-8655, Japan

**Keywords:** Hypoxia-inducible factor 1 (HIF1), HIF-prolyl hydroxylase (HIF-PH) inhibitors, Von Hippel–Lindau (VHL) factor, Cardiac remodelling, Heart failure with preserved ejection fraction (HFpEF)

## Abstract

**Aims:**

Hypoxia-inducible factor (HIF) signalling influences cardiomyocyte differentiation, maturation, and metabolic adaptation under pathological conditions. HIF-Prolyl hydroxylase domain (HIF-PH) inhibitors, which target this pathway, have been introduced for the treatment of renal anaemia. Their precise effect or safety on cardiac function remains unclear because their pharmacokinetics and distribution are not well-understood. This study aimed to examine HIF signalling activation in adult cardiomyocytes (CMs).

**Methods and results:**

We used tamoxifen (TAM)-inducible, CM-specific von Hippel–Lindau (VHL) knockout (VHL-MCM) mice to activate CM HIF signalling. Then we subjected the mice to normal ageing or high-fat diet (HFD) and L-NAME feeding, a murine model of heart failure with preserved ejection fraction (HFpEF). In normal ageing group, there was no difference in the echocardiographic parameters or tissue fibrosis between VHL-MCM and control mice. VHL-MCM mice exhibited significantly increased capillary density and higher expression levels of HIF-target genes (*P* = 0.0248, two-way ANOVA). Under HFD + L-NAME treatment, VHL-MCM mice showed transient but significantly preserved global longitudinal strain (GLS) at 12 weeks post-TAM injection compared to controls (*P* = 0.0284, two-way ANOVA). Sirius red staining indicated a trend towards reduced whole-heart and interstitial fibrosis with significant increase in capillary density in VHL-MCM mice.

**Conclusion:**

Sustained HIF signalling activation in adult CM does not impair the cardiac structure and function in normal ageing process and shows transient yet beneficial effect in murine HFpEF model.

## Graphical Abstract

**Graphical Abstract:**
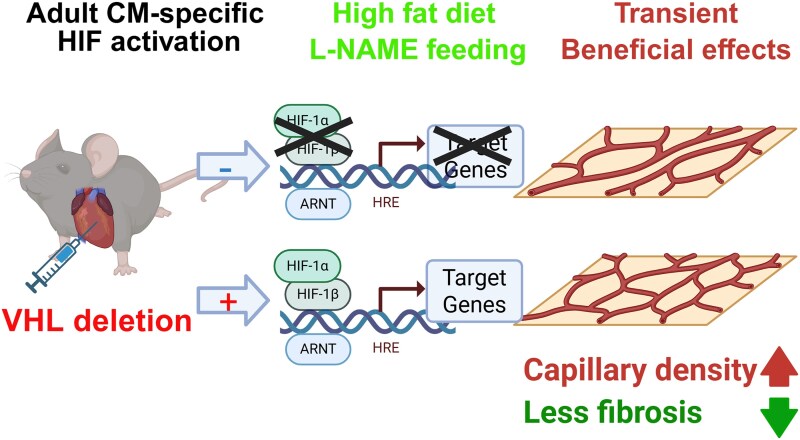

Translational perspectives HIF-PH inhibitors have been widely introduced for the treatment of renal anaemia. However, it is still unclear whether long-term activation of HIF signalling affects cardiac function. We investigated the long-term effects of HIF activation through VHL deletion in mouse cardiomyocytes (CMs). VHL deletion in mature CMs under normal ageing and metabolic overload conditions resulted in prolonged HIF activation, increased capillary density and mitigated CM hypertrophy. This led to transient functional benefits without impairing cardiac function. Our findings provide valuable insights into the potential risks and safety concerns associated with the long-term use of HIF-PH inhibitors.

## Introduction

Hypoxia-inducible factors (HIFs; HIF-1α and HIF-2α) are key regulators of cellular adaptation to hypoxia.^1^ Under normoxic conditions, HIF-prolyl hydroxylase containing-domain protein (HIF-PH) promotes its rapid degradation via von Hippel–Lindau (VHL) factor, thereby preventing nuclear translocation and transcriptional activity.^2^ Under hypoxic conditions, HIF signalling supports cellular and tissue function by promoting angiogenesis via vascular endothelial growth factor (VEGF), shifting metabolism towards glycolysis, and enhancing oxygen transport through erythropoietin.^3^ HIF-PH inhibitors, which stabilize HIF signalling, have been introduced for the treatment of renal anaemia, demonstrating haemoglobin level improvements comparable to erythropoietin therapy in both haemodialysis and non-dialysis patients.^4^ Beyond erythropoiesis, HIF signalling influences multiple myocardial cellular and molecular processes, including cardiomyocyte (CM) differentiation, cardiac tissue maturation, and metabolic adaptation in pathological conditions.^5^ Therefore, precise HIF regulation is essential for balancing myocardial metabolism and maintaining normal cardiac function. It has been reported that activation of CM HIF signalling in developmental stage disrupts cardiac structure and elicits embryonic lethality.^6^ Therefore, it is unknown whether activation of HIF signalling is beneficial for cardiac function.^7^ In order to understand the role of HIF signalling activation in adult CM, we generated the mice with VHL deletion in the adult myocardium by tamoxifen (TAM) -inducible conditional Cre recombination. In this study, we investigated the long-term effects of sustained HIF signalling activation by CM VHL deletion. VHL deletion in mature CMs resulted in prolonged HIF signalling activation under normal ageing and metabolic overload conditions without impairing cardiac function. Additionally, short-term HIF activation increased capillary density and mitigated CM hypertrophy, leading to transient functional benefits. These findings suggest that prolonged HIF signalling exerts transient yet beneficial effects on cardiac function.

## Method

### Mice and experimental design

We purchased VHL flox/flox mice (JAX stock #004081, The Jackson Laboratory) and transgenic mice expressing TAM-inducible, cardiac-specific MerCreMer under the α-myosin heavy chain (αMHC) promoter (A1cfTg(Myh6-cre/Esr1*)1Jmk/J (JAX stock #005657, Myh6-MerCreMer mice), which express TAM-inducible, cardiac-specific MerCreMer under the αMHC promoter. To generate cardiomyocyte-specific inducible VHL knockout mice (VHL-MCM), we crossed Myh6-MerCreMer mice with VHL flox/flox mice. Generated VHL-MCM mice were further mated with C57BL/6J strain mice over 10-genenration and checked genotypes. At 10 weeks of age, we injected VHL-MCM mice with TAM (Sigma-Aldrich) to induce gene recombination. Cre-negative littermates were also injected the same amount of TAM and served as controls. We dissolved tamoxifen in corn oil and administered it via a single intraperitoneal injection (40 mg/kg body weight). This amount can avoid the direct cardiac toxicity of TAM injection according to the previous report.^8^ We housed the mice in a specific pathogen-free facility under a 12-hour light/dark cycle and used 10-week-old male mice for experiments. In the normal ageing study, the mice received a standard chow diet. To induce HFpEF condition, we fed the mice a 70% fat-containing diet (HFD, Research Diets) and supplemented their drinking water with Nω-nitro-L-arginine methyl ester (L-NAME, Sigma-Aldrich) to inhibit constitutive nitric oxide synthase.^9^ We recorded body weight monthly and assessed cardiac function at designated time points using the Vevo2100® Ultrasound System (FujiFilm VisualSonics, Toronto, Canada). All animal experiments followed institutional guidelines and received approval from the Use and Care of Experimental Animals Committee of Jichi Medical University.

### Histological study

Formalin-fixed, paraffin-embedded heart sections were used for histological analysis.

Mouse heart sections were incubated with HIF-1α antibody (NOVUS, NB100-1349). Secondary antibodies conjugated with horseradish peroxidase (Dako) and 3,3′-diamiobenzidine (DAB; Dako) were used to confirm labelling and counterstained with haematoxylin. Cardiomyocyte (CM) nuclei were selected according to the nuclear location and morphology.^10^ CM size was assessed using wheat germ agglutinin (WGA) conjugated with a green fluorescent dye (ThermoFisher). DAPI was used to counterstain nuclei (ThermoFisher). The CM size was quantified with ImageJ (version 2.1.0, NIH, Bethesda, MD, USA). Capillary numbers were counted using isolectin-B4 (IB4) conjugated with a red fluorescent dye (ThermoFisher). The quantification was performed in three randomly selected fields per section and normalized to cell surface area. Fluorescent images were captured using an FSX-100 inverted microscope (OLYMPUS, Tokyo, Japan). Sirius Red and Fast Green staining were used to evaluate whole-heart tissue and interstitial fibrosis. For interstitial fibrosis evaluation, we used high magnification (×200) images and selected the area where CM aligns in a longitudinal way. We cropped and removed the apparent perivascular fibrotic area, and quantified remaining fibrotic (red) area. The quantification was performed in three to five randomly selected fields per section and normalized to tissue surface area. Bright-field images were obtained with the VS120 virtual slide system (OLYMPUS, Tokyo, Japan), and fibrotic areas were quantified using ImageJ (version 2.1.0, NIH, Bethesda, MD, USA).

### RNA isolation, reverse transcription, and quantitative real-time polymerase chain reaction (qPCR)

Total RNA was extracted from mouse hearts using the NucleoSpin RNA kit (Takara Bio, Shiga, Japan). Complementary DNA was synthesized from RNA templates using ReverTra Ace reverse transcriptase (TOYOBO, Osaka, Japan). Quantitative PCR was performed using THUNDERBIRD SYBR qPCR Mix (TOYOBO, Osaka, Japan) with the Roche LightCycler 480. The relative standard curve method was used for quantification, with results normalized to Glucuronidase Beta (Gusb) expression. Targeted genes were *Vgefa*, *Lactate dehydrogenase A (Ldha), Phosphoglycerate kinase 1 (Pgk1)*, and *Solute carrier family 2 member 1 (Slc2a1*). Primer sequences used in this study are listed in *Table 3*.

### Statistical analyses

Data are presented as mean ± standard error of the mean (SEM). Statistical significance was determined using the Welch's *t*-test and one-way/two-way ANNOVA with *P*-values < 0.05 considered significant. Analyses were performed using GraphPad Prism 9.0 (GraphPad Software, San Diego, CA, USA).

## Results

### Deletion of VHL in adult cardiomyocytes upregulates HIF-1α and HIF target gene expression

**Figure 1:**
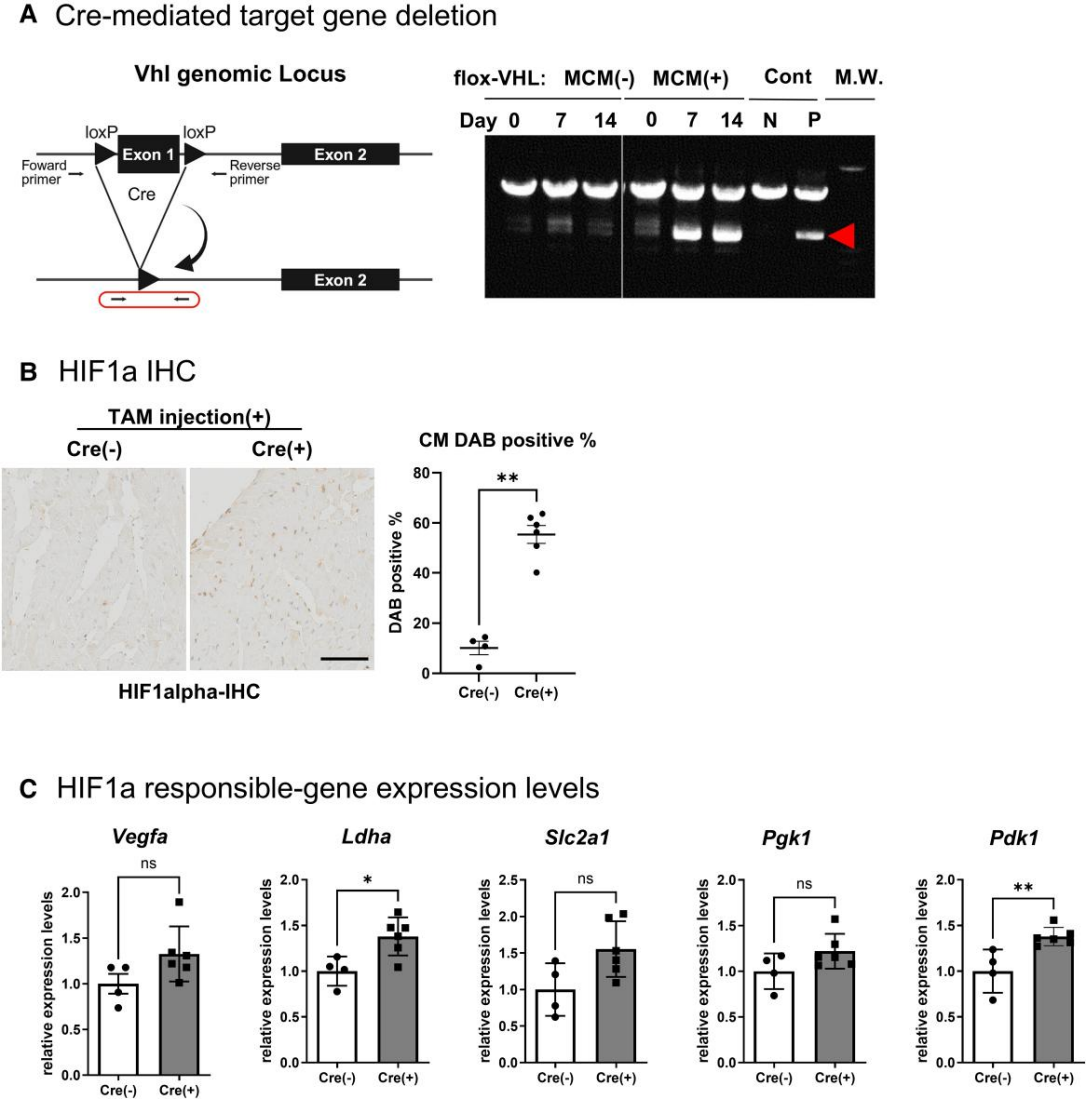
VHL deletion resulted in up-regulation of HIF1 responsible genes. (*A*) Competitive PCR analysis of the floxed *Vhl* loci in myocardium from adult VHL flox/flox (VHL-MCM-) and VHL flox/flox; MCM (VHL-MCM+) mice. The diagram on the left shows the deletion target sequence (exon 1) on the VHL gene, as well as the positions of the PCR primers. The region enclosed by the line corresponds to the PCR product indicated by the arrowhead in the PCR result panel on the right. Abbreviations; Cont: control, N: negative control, P: positive control, and M.W.: molecular weight marker. (*B*) Immunostaining of HIF-1α in isolated hearts from VHL-MCM-: Cre^–^ or VHL-MCM: Cre (+) mice. Brown-stained nuclei (DAB-positive) are the HIF-1α-positive cells. Dot graph indicates the quantification of DAB-positive nuclear cardiomyocyte proportions. (*C*) Gene expression analysis. Quantitative RT-PCR measured mRNA levels of HIF-1α target genes in VHL-MCM (*n* = 6) and control (*n* = 4) hearts after 14 days TAM injection. (*B*), (*C*), ns: no significant, *P < 0.05, **0.01 < *P* < 0.005 by un-paired *t* test.

To induce cardiac-specific HIF signalling activation, we generated inducible VHL knockout mice with cardiomyocyte-specific expression (VHL-MCM). We confirmed effective deletion of exon 1 of the *Vhl* gene by competitive PCR after TAM treatment (*Figure 1A*). Immunostaining of HIF-1α confirmed significantly elevated nuclear protein levels in cardiomyocytes (*Figure 1B*). Furthermore, the gene expression levels of HIF-target genes were clearly upregulated 14 days after TAM injection (*Figure 1C*).

### Matured CM VHL deletion shows transient LV GLS improvement under HFpEF conditions

**Figure 2:**
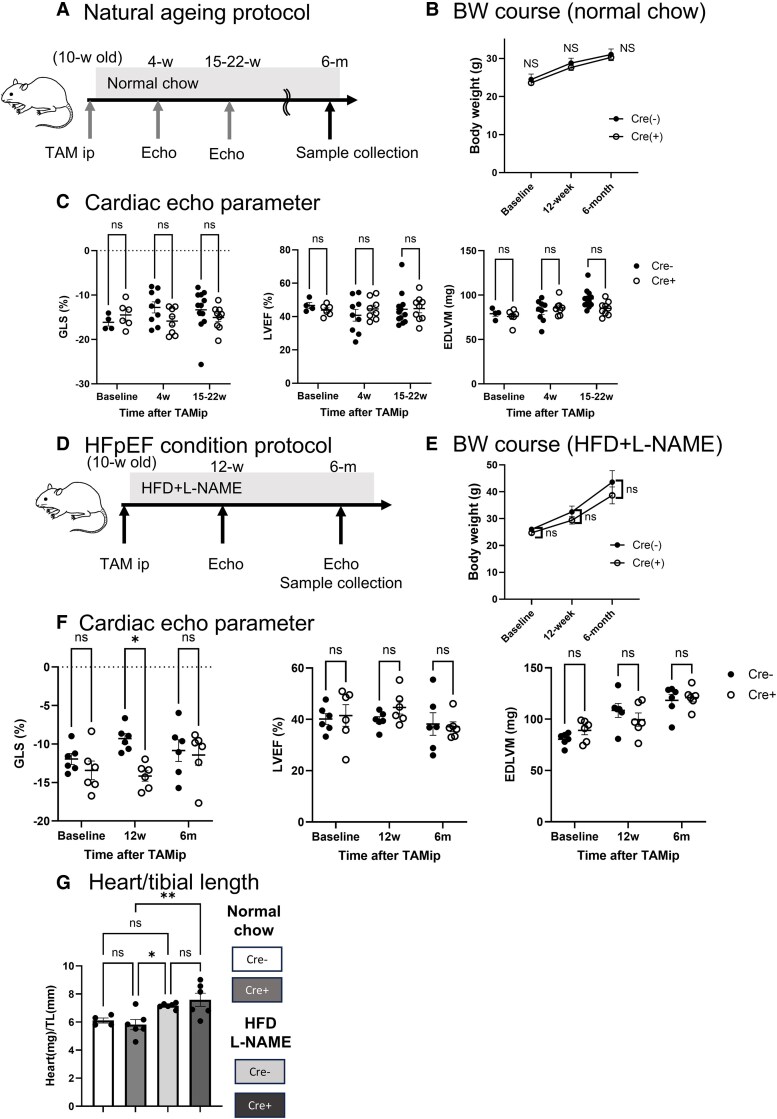
Matured CM VHL deletion shows transient LV GLS improvement under HFpEF condition. (*A*) Experimental design of natural ageing model (normal chow diet). Tamoxifen (TAM) treatment at 10 weeks induced *Vhl* deletion in cardiomyocyte-specific conditional knockout mice (VHL-MCM; Cre+). Cre-negative littermate controls (Cre−) received the same treatment. Echocardiography evaluated cardiac function at 4-week (4-w) and 15–22-week (12–22-w) post-injection. Cardiac tissue was analysed at 6 months (6 m). (*B*) Body weight measurements (normal chow diet). No significant differences were observed between VHL-MCM (*n* = 6) and control (*n* = 4) mice. (*C*) Echocardiographic assessment (normal chow diet). Longitudinal evaluation of cardiac function in VHL-MCM (*n* = 6–9) and control (*n* = 4–12) mice. (*D*) Experimental design of HFpEF condition (HFD + L-NAME feeding). TAM treatment at 10 weeks induced *Vhl* deletion in cardiomyocyte-specific conditional knockout (VHL-MCM, Cre+) mice. Cre-negative littermate controls (Cre−) received the same amount of TAM injection. VHL-MCM and control mice were maintained on a high-fat diet (HFD; 60% kilocalories from fat) and L-NAME (0.5 g/L in drinking water) for 6 months. Echocardiography evaluated cardiac function at 12-week (12-w) and 6-month (6-m) post-diet initiation. Cardiac tissue analysis was performed at 6 months. (*E*) Body weight measurements (HFD + L-NAME feeding). No significant differences were observed between VHL-MCM (*n* = 12) and control (*n* = 11) mice. (*F*) Echocardiographic assessment (HFD + L-NAME feeding). Longitudinal evaluation of cardiac function in VHL-MCM (Cre+, *n* = 6) and control (Cre−, *n* = 6) mice. (*G*) Heart weight (mg) standardized by tibial length (mm) at organ sample collection from VHL-MCM (*n* = 6) and control (*n* = 4) mice at 6 months with normal chow, and VHL-MCM (Cre+, *n* = 6) and control (Cre−, *n* = 6) mice at 6 months with HFD + L-NAME feeding. (*B*), (*C*), (*E*), (*F*) ns: no significant, *P < 0.05, by two-way ANNOVA with Tukey *post hoc* analysis. (*G*) ns: no significant, *P < 0.05, **0.01 < *P* < 0.005, by one-way ANNOVA with Tukey *post hoc* analysis.

To examine the influence of adult cardiomyocyte-specific HIF signalling activation on cardiac structure and function, we injected TAM into VHL-MCM mice and Cre-negative littermate controls and maintained them on a standard chow diet for 6 months. Cardiac echography was performed at baseline and at 4 weeks, 15–22 weeks, and 6 months post-TAM injection. Cardiac tissue was collected at 6 months for histological and gene expression analyses (*Figure 2A*). There was no significant difference in body weight between the groups (*Figure 2B*). Echocardiography revealed no significant changes in global longitudinal strain (GLS), left ventricular ejection fraction (LVEF), or end-diastolic left ventricular mass (EDLVM) throughout the study period (*Figure 2C* and *Table 1*). To further evaluate the effects of HIF activation in CM under pathological conditions, we fed VHL-MCM mice a HFD and L-NAME (HFD + L-NAME), a two-hit murine model of HFpEF. We administered TAM at 10 weeks of age and maintained the mice on an HFD + L-NAME diet for 6 months. We performed echocardiography at baseline and at 12 weeks and 6 months after TAM treatment, followed by tissue collection at the study endpoint (*Figure 2D*). There was no difference in weight gain between the two groups when fed an HFD + L-NAME for 6 months. (*Figure 2E*). While GLS declined significantly in control mice, VHL-MCM mice maintained preserved GLS 12 weeks after being fed an HFD + L-NAME diet. However, GLS values were quite variable among the animals at 6 months, and there was no clear difference between the groups. Echocardiography revealed that LV mass and heart weight increased with HFD + L-NAME in both groups, but there were no differences in the parameters (*Figure 2F & G*, and *Table 2*).

**Table 1:** Cardiac echo parameters with normal chow diet for 6 months

		Normal chow														
		Baseline					4-week					15–22-week				
		Cre(−)		Cre(+)			Cre(−)		Cre(+)			Cre(−)		Cre(+)		
		Average	SD	Average	SD	*P*-value	Average	SD	Average	SD	*P*-value	Average	SD	Average	SD	*P*-value
EDV	μL	58.7	4.9	58.2	8.4	0.9016	58.1	15.5	60.4	5.9	0.6845	64.2	8.3	67.2	10.1	0.4802
ESV	μL	31.2	3.7	32.4	4.1	0.6372	35.1	12.5	33.3	5.5	0.7139	36.2	9.5	37.1	8.0	0.8245
SV	μL	27.6	2.7	25.8	4.9	0.4873	23.0	7.0	27.1	4.3	0.1675	28.0	3.4	30.1	6.6	0.3969
FS	%	19.7	3.2	20.2	3.7	0.8213	20.5	8.8	20.1	4.3	0.8887	21.3	6.7	22.0	4.5	0.7594
CO	mL/min	13.5	0.7	13.1	2.7	0.4037	10.9	3.7	13.6	2.5	0.1034	14.2	2.4	15.3	3.8	0.4683
GLS	%	−16.1	1.7	−14.5	2.8	0.2801	−12.8	3.7	−15.9	2.9	0.0784	−13.3	4.7	−15.1	2.7	0.2984
EDLVM	mg	79	6	76	8	0.488	82	12	86	8	0.5078	96	11	85	8	0.0142
ESLVM	mg	83	5	78	8	0.2488	85	13	89	8	0.5389	98	11	87	8	0.0139
Heart Rate	bpm	492	25	506	18	0.3724	467	53	501	38	0.1534	505	39	505	37	0.9986
EF	%	46.6	3.7	43.9	3.3	0.2742	40.7	10.6	44.6	6.3	0.3775	44.3	10.0	44.7	7.4	0.9201

Table 1 shows in detailed cardiac echo parameters from control (Cre-) and VHL-MCM (Cre+) mice with normal chow diet for 6-month

EDV: end-diastolic volume, ESV: end-systolic volume, SV: stroke volume, FS: fractional shortening, CO: cardiac output, GLS: global longitudinal strain, EDLVM: end-diastolic left ventricular mass, ESLVM: end-systolic left ventricular mass, EF: ejection fraction, SD: standard deviation. *P*-values are statistical significance by un-paired t test.

**Table 2:** Cardiac echo parameters with high fat diet and L-NAME feeding for 6 months

		HFD + L-NAME														
		Baseline					4-week					15–22-week				
		Cre(−)		Cre(+)			Cre(−)		Cre(+)			Cre(−)		Cre(+)		
		Average	SD	Average	SD	*P*-value	Average	SD	Average	SD	*P*-value	Average	SD	Average	SD	*P*-value
EDV	μL	54.7	5.8	62.9	7.5	0.0615	58.5	6.5	65.3	15.4	0.353	59.9	9.8	66.7	10.0	0.264
ESV	μL	32.8	5.7	36.6	7.9	0.3656	35.3	5.0	36.5	11.1	0.8121	37.4	11.1	41.6	6.4	0.4375
SV	μL	21.9	2.1	26.3	7.5	0.2146	23.2	2.4	28.8	5.3	0.0523	22.6	5.4	25.0	5.7	0.4591
FS	%	21.4	5.1	21.3	8.0	0.9616	20.6	5.0	20.9	3.2	0.9017	16.9	7.4	16.6	1.8	0.9325
CO	mL/min	9.6	1.5	13.4	3.5	0.0474	12.6	1.3	15.7	3.2	0.0683	12.6	3.5	13.9	3.9	0.5496
GLS	%	−11.9	1.8	−13.4	3.0	0.3215	−9.3	1.6	−14.2	1.6	0.0004	−10.8	3.5	−11.4	3.3	0.7727
EDLVM	mg	80	6	89	10	0.1201	109	17	100	16	0.3617	118	14	121	10	0.7207
ESLVM	mg	82	8	92	12	0.1255	112	18	102	16	0.3635	120	14	124	9	0.5886
Heart Rate	bpm	438	35	514	31	0.1283	546	26	546	18	0.995	556	21	552	33	0.8128
Ef	%	40.1	5.2	41.5	10.3	0.7795	39.7	3.3	44.7	6.3	0.1299	38.4	12.1	37.1	4.7	0.8328

*Table 2* indicates parameters from control (Cre-) and VHL-MCM (Cre+) mice with high fat diet and L-NAME feeding for 6 months

EDV: end-diastolic volume, ESV: end-systolic volume, SV: stroke volume, FS: fractional shortening, CO: cardiac output, GLS: global longitudinal strain, EDLVM: end-diastolic left ventricular mass, ESLVM: end-systolic left ventricular mass, EF: ejection fraction, SD: standard deviation. *P*-values are statistical significance by un-paired *t* test.

**Table 3:** Quantitative PCR primer sets

Primer	Sequence
Vegfa Forward	5′-GGAGAGCAGAAGTCCCATGA-3′
Vegfa Reverse	5′-ACTCCAGGGCTTCATCGTTA-3′
Slc2a1 Forward	5′-CTGGACGACGGACCCTGC-3′
Slc2a1 Reverse	5′-AAAGAAGGCCACAAAGCCAAAG-3′
Pgk1 Forward	5′-CTGTGGTACTGAGAGCAGCAAGA-3′
Pgk1 Reverse	5′-CAGGACCATTCCAAACAATCTG-3′
Pdk4 Forward	5′-TGGAGTTCCATGAGAAGAGC-3′
Pdk4 Reverse	5′-TGACCAGCGTGTCTACAAAC-3′
Hmgcs2 Forward	5′-TCATTCCGAGTGTCCAAGGA-3′
Hmgcs2 Reverse	5′-TCTGACACACTAGACACCAGT-3′
Acot1 Forward	5′-GAAACCATGCACATGGAGTAC-3′
Acot1 Reverse	5′-CATAGCAAGGCCAAGTTCAC-3′
Gusb Forward	5′-AAAATCACCCTGCGGTTGT-3′
Gusb Reverse	5′-TGTGGGTGATCAGCGTCTT-3′

*Table 3* indicates used primer sets for qPCR.

### Matured CM with VHL deletion shows suppressed fibrotic changes, a modified CM size distribution, and preserved myocardial capillary density under HFpEF conditions

**Figure 3:**
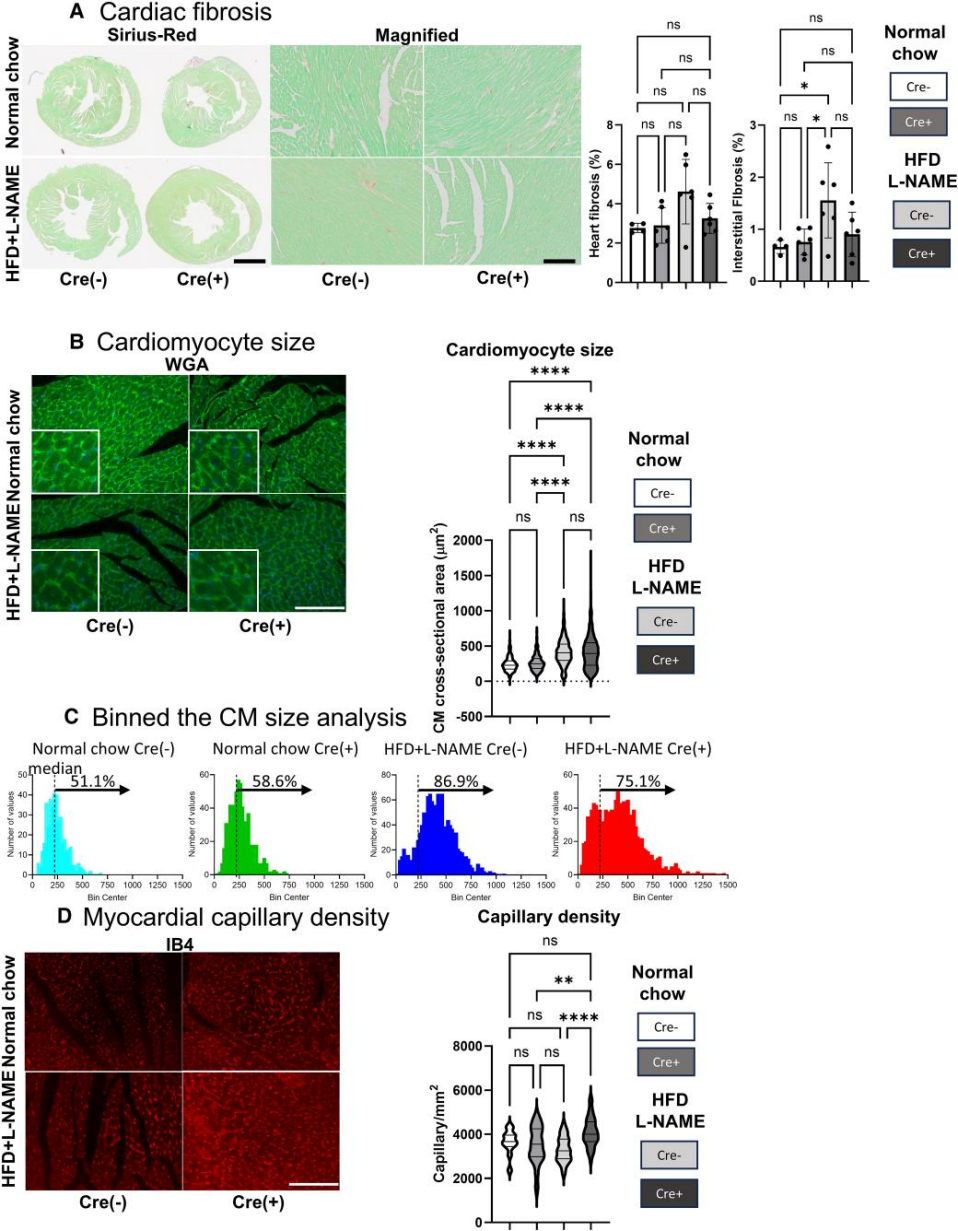
Matured CM VHL deletion shows a trend towards reduced cardiac fibrosis with significant increase in capillary density and mitigated CM hypertrophy under HFpEF condition. (*A*) Fibrosis quantification by Sirius-Red staining. Left panels are representative low and high magnified pictures from 4 groups: VHL-MCM (*n* = 6) and control (*n* = 4) mice at 6 months with normal chow, and VHL-MCM (Cre+, *n* = 6) and control (Cre−, *n* = 6) mice at 6 months with HFD + L-NAME feeding. Scale bar = 1 mm and 50 µm. Right dot graphs are fibrotic area quantification and comparison among 4 groups (Heart fibrosis and interstitial fibrosis). (*B*) Cardiomyocyte (CM) size analysis. CM cross-sectional area was quantified by wheat germ agglutinin (WGA) staining. Left panels are representative low and high magnified (inserts) pictures from four groups: VHL-MCM (*n* = 6) and control (*n* = 4) mice at 6 months with normal chow, and VHL-MCM (Cre+, *n* = 6) and control (Cre−, *n* = 6) mice at 6 months with HFD + L-NAME feeding. Right violin-plot graphs are CM size distribution and comparison among 4 groups. Scale bar = 50 µm. (*C*) Binned the cardiomyocyte (CM) cross-sectional area analysis. We further analysed the CM size distribution and difference. According to the cut-off CM size (221 μm^2^, a median value of CMs from Cre (−) mice with normal chow for 6 months), we showed the CM size distributions of the four groups and indicated the percentage of the larger CM proportions above the cut-off value. (*D*) Myocardial capillary density analysis. Capillary density was quantified by isolectin-B4 (IB4) staining. Left panels are representative pictures from 4 groups: VHL-MCM (*n* = 6) and control (*n* = 4) mice at 6 months with normal chow, and VHL-MCM (Cre+, *n* = 6) and control (Cre−, *n* = 6) mice at 6 months with HFD + L-NAME feeding. Right violin-plot graphs are capillary density distributions and comparison among 4 groups. Scale bar = 50 µm. (*A*), (*B*), (*D*), ns: no significant, *P < 0.05, **0.01 < *P* < 0.005, ****P < 0.0001 by one-way ANOVA with Tukey *post hoc* analysis.

To analyse the effect of VHL deletion on cardiac fibrosis, we subjected four groups of myocardial samples to Sirius red staining (*Figure 3A*). A 6-month normal chow diet showed no significant changes in whole-heart or interstitial fibrosis in VHL-MCM and control mice. An HFD + L-NAME feeding increased fibrosis levels compared to chow-fed mice, though there were no significant differences between VHL-MCM and control mice (*Figure 3A*, right dot graphs).

To evaluate the change in CM size due to VHL deletion, we performed WGA staining on the four groups of samples. There was no significant difference between VHL-MCM and control mice after a 6-month normal diet. HFD + L-NAME feeding for 6 months significantly increased CM size in both VHL-MCM and control mice (*Figure 3B*). Although the overall CM size remained comparable, the size distribution differed. Control mice that received a 6-month HFD + L-NAME diet displayed a clearly higher proportion of larger CMs (*Figure 3C*).

To further investigate the effects of CM-VHL deletion on capillary rarefaction, we compared capillary densities among four groups (*Figure 3D*). There was no significant difference in capillary density between VHL-MCM and control mice with normal chow. However, VHL-MCM myocardium after 6 months of an HFD + L-NAME feeding showed significantly higher capillary density compared to control mice.

### Matured CM VHL deletion shows an upward trend in HIF-target gene expression under normal chow and HFD + L-NAME feeding

**Figure 4:**
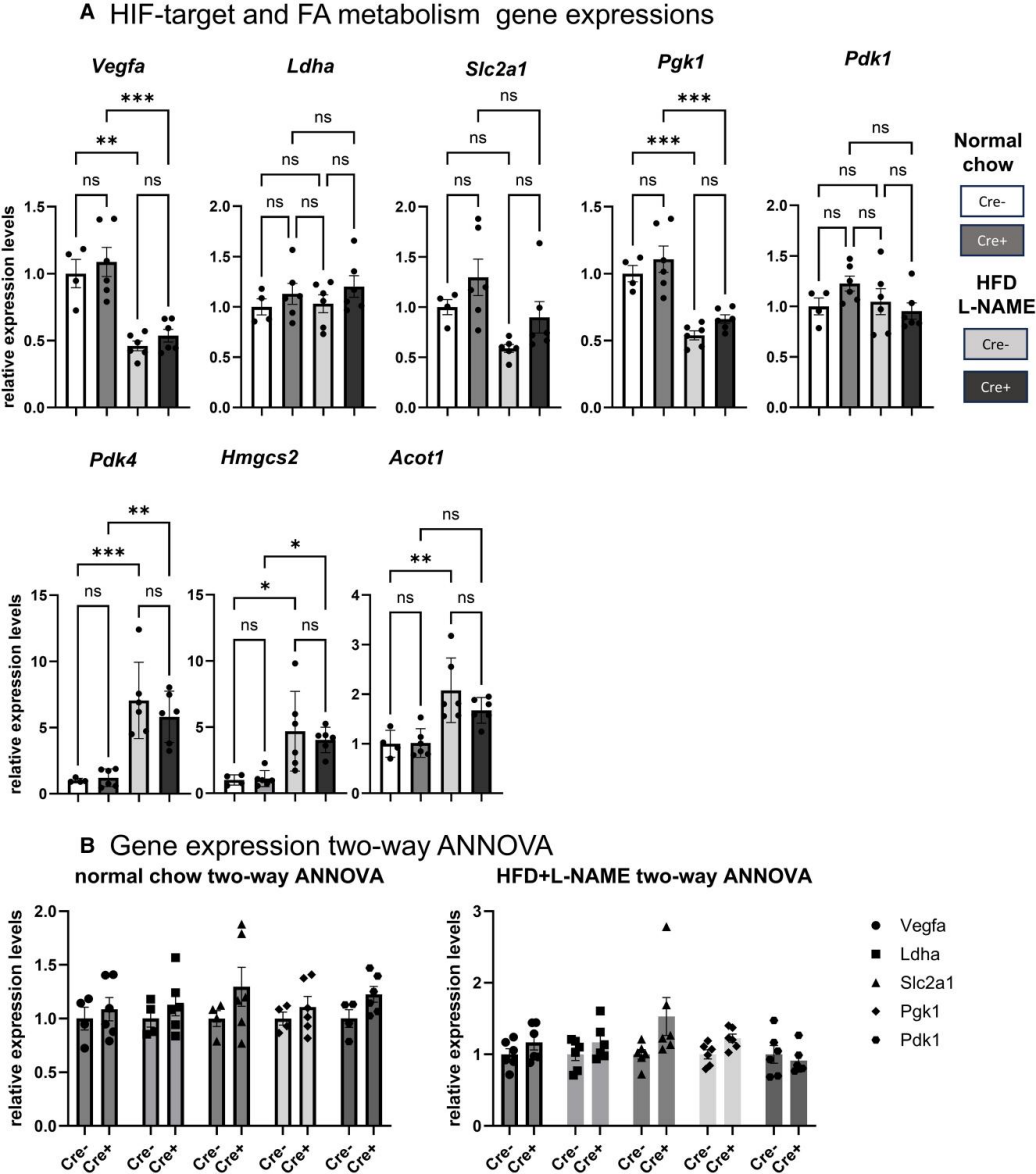
Matured CM VHL deletion shows an upward trend in HIF-target gene expression under normal chow and HFD + L-NAME feeding. (*A*) Gene expression analysis. Quantitative RT-PCR measured mRNA levels of HIF-1α target genes and fatty acid utilization-related genes in hearts from VHL-MCM (*n* = 6) and control (*n* = 4) mice at 6 months with normal chow, and VHL-MCM (Cre+, *n* = 6) and control (Cre−, *n* = 6) mice at 6 months with HFD + L-NAME feeding. (*B*) Relative gene expression graphs used for two-way ANOVA statistical analysis to validate the HIF-target gene expression trend between control (Cre-) and VHL-MCM (Cre+) mice with 6-month normal chow diet or HFD + L-NAME feeding. (*A*) ns: no significant, *P < 0.05, **0.01 < *P* < 0.005, ***0.0001 < *P* < 0.005, by one-way ANNOVA with Tukey *post hoc* analysis. (*B*) two-way ANNOVA with Tukey *post hoc* analysis.

Finally, we carried out a gene expression analysis using myocardial samples from the four groups to evaluate the expression of HIF-target: *Vegfa*, *Slc2a1*, *Ldha*, *Pgk1*, *Pdk1*, and fatty acid metabolism-related genes: *Pdk4*, *Hmgcs*, and *Acot1* (*Figure 4A*).

Gene expression analysis revealed an upward trend in HIF-target gene expression in VHL-MCM mice under normal chow (*P* = 0.0248, two-way ANOVA) and HFD + L-NAME feeding (*P* = 0.0120, two-way ANOVA; *Figure 4B*). Compared to the chow-fed condition, the HFD + L-NAME diet markedly increased FA utilization gene expression and reduced HIF-target gene expression, potentially minimizing differences in HIF-related gene expression between groups.

## Discussion

The long-term effects of HIF activation are still controversial. Even after HIF-PH inhibitors were introduced to treat renal anaemia, concerns about potential adverse effects remain, particularly regarding malignancies and VEGF-mediated growth promotion. There have also been suggestions of cardiovascular risks based on reports linking modulation of the HIF pathway to impaired cardiac development and function. In our study, we activated HIF signalling in adult cardiomyocytes (CMs) and assessed its effects on cardiac structure and function in normal ageing and heart failure with preserved ejection fraction (HFpEF) models. Notably, long-term HIF upregulation did not lead to functional decline or increased mortality. Under metabolic stress, VHL-MCM mice exhibited reduced pathological myocardial remodelling, including reduced fibrosis and increased capillary density. Activation of the HIF pathway promoted these initial structural and functional benefits, which persisted for up to 12 weeks after TAM injection.^11^ An increase in capillary density may underlie the beneficial effects of HIF activation in VHL-MCM mice.^12^

Various factors, such as adenosine, neuronal influences, CO₂, O₂, pH, lactate, K⁺, myogenic responses, growth factors, NO, and others, have been suggested to regulate cardiac blood flow.^13^ However, these numerous phenomena cannot be explained by a single factor or signalling pathway.^14^ Unlike healthy hearts, hearts subjected to a HFD and L-NAME are exposed to various stresses, such as stretching, metabolic stress, and oxidative stress. Our findings suggest that enhancing cardiac VEGF signalling alone is insufficient to induce angiogenesis under control conditions. However, mechanical and metabolic stresses present in the HFD/L-NAME model are also important for angiogenesis.

In fact, oxygen supplied by the cardiac vessels is used to produce ATP. The HFD/L-NAME model consumes more ATP and oxygen than healthy hearts, suggesting the presence of organizational factors that promote angiogenesis.^15,16^ Additionally, balancing the energy fuel in CM may affect cardiac remodelling in the HFpEF model since excess fatty acid utilization could lead to cardiac dysfunction.^17^ Further studies are needed to fully elucidate the effects of glucose metabolism in the HFpEF model.

### Limitations

This study has certain limitations. An important consideration in our research is the use of the C57BL/6J strain. Recent reports suggest that this strain exhibits a weaker HFpEF phenotype in the two-hit model, including limited diastolic dysfunction.^18^ While our two-hit HFpEF model shows the phenotypic requirements of obesity, glucose metabolism abnormalities, cardiac remodelling (hypertrophy and fibrosis) and dysfunction (declined GLS), it cannot be ruled out that this may impact our study's objective of confirming the long-term effects of VHL deletion in cardiomyocytes. To fully clarify strain differences, head-to-head comparisons across different studies are necessary. Caution should be exercised when selecting strains for intervention studies targeting more severe heart failure with diastolic dysfunction by the two-hit HFpEF model or for research targeting pathways in which differences between the C57BL/6J and 6N strains are already evident, such as the nicotinamide nucleotide transhydrogenase (NNT) pathway.^19,20^

Myocardial tissue is composed of multiple cell types, including fibroblasts, vascular cells, and immune cells. HIF-PH inhibitors could affect each of these cell types differently. To comprehensively evaluate the effects of the HIF pathway on long-term tissue remodelling, it is essential to consider the contributions of each cell type and the interactions between them. Understanding the different cellular responses to HIF modulation is critical to evaluating the therapeutic potential of HIF targeting.

## Lead author biography


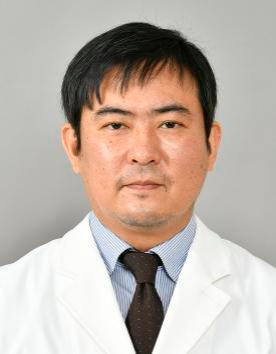


Daigo Sawaki is an MD in cardiac diseases and PhD in cardiovascular physiology. He is an associate professor of community and family medicine, clinical pharmacology, and cardiology (Jichi Medical University). From 2014-2020, he belonged to Professor Derumeaux’s laboratory (University of Paris Est-Creteil) as an associate professor, and engaged research work on cardiometabolic-senescence pathologies with numerous publications in high-impact journals. Since 2020, he coordinates the basic and clinical research focusing on senescence-related cardiomyopathy such as transthyretin cardiomyopathy. His research activity has been entirely dedicated to deciphering the pathophysiology of cardiac remodelling.

## Data Availability

The data that support the findings of this study are available from the authors upon reasonable request.

## References

[1] Semenza GL . Hypoxia-inducible factors in physiology and medicine. Cell 2012;148:399–408. 22304911 10.1016/j.cell.2012.01.021 PMC3437543

[2] Kaelin WG, Ratcliffe PJ. Oxygen sensing by metazoans: the central role of the HIF hydroxylase pathway. Mol Cell 2008;30:393–402. 18498744 10.1016/j.molcel.2008.04.009

[3] Haase VH . Regulation of erythropoiesis by hypoxia-inducible factors. Blood Rev 2013;27:41–53. 23291219 10.1016/j.blre.2012.12.003 PMC3731139

[4] Chertow GM, Pergola PE, Farag YMK, Agarwal R, Arnold S, Bako G, Block GA, Burke S, Castillo FP, Jardine AG, Khawaja Z, Koury MJ, Lewis EF, Lin T, Luo W, Maroni BJ, Matsushita K, McCullough PA, Parfrey PS, Roy-Chaudhury P, Sarnak MJ, Sharma A, Spinowitz B, Tseng C, Tumlin J, Vargo DL, Walters KA, Winkelmayer WC, Wittes J, Eckardt K-U. Vadadustat in patients with anemia and non-dialysis-dependent CKD. N Engl J Med 2021;384:1589–1600. 33913637 10.1056/NEJMoa2035938

[5] Natarajan R, Salloum FN, Fisher BJ, Kukreja RC, Fowler AA. Hypoxia inducible factor-1 activation by prolyl 4-hydroxylase-2 gene silencing attenuates myocardial ischemia reperfusion injury. Circ Res 2006;98:133–140. 16306444 10.1161/01.RES.0000197816.63513.27

[6] Menendez-Montes I, Escobar B, Palacios B, Gómez MJ, Izquierdo-Garcia JL, Flores L, Jiménez-Borreguero LJ, Aragones J, Ruiz-Cabello J, Torres M, Martin-Puig S. Myocardial VHL-HIF signaling controls an embryonic metabolic switch essential for cardiac maturation. Dev Cell 2016;39:724–739. 27997827 10.1016/j.devcel.2016.11.012

[7] Schiattarella GG, Altamirano F, Tong D, French KM, Villalobos E, Kim SY, Luo X, Jiang N, May HI, Wang ZV, Hill TM, Mammen PPA, Huang J, Lee DI, Hahn VS, Sharma K, Kass DA, Lavandero S, Gillette TG, Hill JA. Nitrosative stress drives heart failure with preserved ejection fraction. Nature 2019;568:351–356. 30971818 10.1038/s41586-019-1100-z PMC6635957

[8] Bersell K, Choudhury S, Mollova M, Polizzotti BD, Ganapathy B, Walsh S, Wadugu B, Arab S, Kühn B. Moderate and high amounts of tamoxifen in αMHC-MerCreMer mice induce a DNA damage response, leading to heart failure and death. Dis Model Mech 2013;6:1459–1469. 23929941 10.1242/dmm.010447 PMC3820268

[9] Chaudhary R, Suhan TK, Wu C, Alzamrooni A, Madamanchi N, Abdel-Latif A. Housing temperature influences metabolic phenotype of heart failure with preserved ejection fraction in J vs N strain C57BL/6 mice. Mol Cell Endocrinol 2025;598:112457. 39788312 10.1016/j.mce.2025.112457 PMC11820722

[10] Droste P, Wong DWL, Hohl M, von Stillfried S, Klinkhammer BM, Boor P. Semiautomated pipeline for quantitative analysis of heart histopathology. J Transl Med 2023;21:666. 37752535 10.1186/s12967-023-04544-2 PMC10523682

[11] Hölscher M, Silter M, Krull S, von Ahlen M, Hesse A, Schwartz P, Wielockx B, Breier G, Katschinski DM, Zieseniss A. Cardiomyocyte-specific prolyl-4-hydroxylase domain 2 knock out protects from acute myocardial ischemic injury. J Biol Chem 2011;286:11185–11194. 21270129 10.1074/jbc.M110.186809 PMC3064173

[12] Shridhar P, Glennon MS, Pal S, Waldron CJ, Chetkof EJ, Basak P, Clavere NG, Banerjee D, Gingras S, Becker JR. MDM2 regulation of HIF signaling causes microvascular dysfunction in hypertrophic cardiomyopathy. Circulation 2023;148:1870–1886. 37886847 10.1161/CIRCULATIONAHA.123.064332 PMC10691664

[13] Goodwill AG, Noblet JN, Sassoon D, Fu L, Kassab GS, Schepers L, Herring BP, Rottgen TS, Tune JD, Dick GM. Critical contribution of KV1 channels to the regulation of coronary blood flow. Basic Res Cardiol 2016;111:56. 27496159 10.1007/s00395-016-0575-0 PMC5193223

[14] Johnson NP, Gould KL, De Bruyne B. Autoregulation of coronary blood supply in response to demand: JACC review topic of the week. J Am Coll Cardiol 2021;77:2335–2345. 33958131 10.1016/j.jacc.2021.03.293

[15] Abe H, Takeda N, Isagawa T, Semba H, Nishimura S, Morioka MS, Nakagama Y, Sato T, Soma K, Koyama K, Wake M, Katoh M, Asagiri M, Neugent ML, Kim J-w, Stockmann C, Yonezawa T, Inuzuka R, Hirota Y, Maemura K, Yamashita T, Otsu K, Manabe I, Nagai R, Komuro I. Macrophage hypoxia signaling regulates cardiac fibrosis via oncostatin M. Nat Commun 2019;10:2824. 31249305 10.1038/s41467-019-10859-w PMC6597788

[16] Shehadeh LA, Robleto E, Lopaschuk GD. Cardiac energy substrate utilization in heart failure with preserved ejection fraction: reconciling conflicting evidence on fatty acid and glucose metabolism. Am J Physiol Heart Circ Physiol 2025;328:H1267–H1H95. 40251758 10.1152/ajpheart.00121.2025 PMC12355381

[17] Finck BN, Lehman JJ, Leone TC, Welch MJ, Bennett MJ, Kovacs A, Han X, Gross RW, Kozak R, Lopaschuk GD, Kelly DP. The cardiac phenotype induced by PPARalpha overexpression mimics that caused by diabetes mellitus. J Clin Invest 2002;109:121–130. 11781357 10.1172/JCI14080 PMC150824

[18] Jacobsen JCB, Schubert IH, Larsen K, Terzic D, Thisted L, Thomsen MB. Preload dependence in an animal model of mild heart failure with preserved ejection fraction (HFpEF). Acta Physiol (Oxf) 2024;240:e14099. 38230889 10.1111/apha.14099

[19] Pepin ME, Konrad PJM, Nazir S, Bazgir F, Maack C, Nickel A, Gorman JM, Hohl M, Schreiter F, Dewenter M, de Britto Chaves Filho A, Schulze A, Karlstaedt A, Frey N, Seidman CE, Seidman JG, Backs J. Mitochondrial NNT promotes diastolic dysfunction in cardiometabolic HFpEF. Circ Res 2025;136:1564–1578.40340422 10.1161/CIRCRESAHA.125.326154

[20] Mekada K, Yoshiki A. Substrains matter in phenotyping of C57BL/6 mice. Exp Anim 2021;70:145–160. 33441510 10.1538/expanim.20-0158 PMC8150240

